# SIRT3 is required for the protective function of ketogenic diet on neural inflammation and neuropathic pain

**DOI:** 10.7150/ijbs.110921

**Published:** 2025-04-21

**Authors:** Mengqiu Deng, Yuanyuan Fang, Yan Chu, Ruifeng Ding, Xiaoyi Fan, Huawei Wei, Honghao Song, Guowei Jiang, Hailing Zhang, Chaofeng Han, Hongbin Yuan

**Affiliations:** 1 Department of Anesthesiology, Changzheng Hospital, Second Affiliated Hospital of Naval Medical University, Shanghai 200003, China.; 2 Department of Anesthesiology, the 940th Hospital of Joint Logistics Support Force of Chinese People's Liberation Army, Lanzhou, Gansu 730050, China.; 3 Department of Anesthesiology, No. 971 Hospital of the People's Liberation Army Navy, Qingdao, Shandong 266071, China.; 4 Department of Neurology, Changhai Hospital, Naval Medical University, Shanghai, China.; 5 Department of Histology and Embryology, National Key Laboratory of Immunity and Inflammation, Naval Medical University, Shanghai 200433, China.

**Keywords:** neuropathic pain, ketogenic diet, sirtuin 3, microglia, β-hydroxybutyrate

## Abstract

Chronic neuroinflammation is a key pathological feature of neuropathic pain. The ketogenic diet (KD) has demonstrated potential to reduce neuronal excitability and alleviate inflammation in epilepsy, yet its effects and precise mechanisms in neuropathic pain remain elusive. We first observed that β-hydroxybutyrate (BHB), a key metabolite induced by KD, was reduced in mice following neuropathic pain induced by chronic constriction injury (CCI). Subsequently, we demonstrated that KD effectively alleviated CCI-induced thermal hyperalgesia and mechanical allodynia, while mitigating neuroinflammation through reduced microglial activation and pro-inflammatory cytokine levels. BHB reduced reactive oxygen species (ROS) production, which coincided with enhanced mitochondrial membrane potential in microglia, thereby attenuating microglia-mediated inflammatory responses. Both *in vivo* and *in vitro* experiments revealed KD-induced upregulation of uncoupling protein 2 (UCP2), sirtuin 3 (SIRT3) and peroxisome proliferator-activated receptor gamma coactivator 1 alpha (PGC-1α) in the spinal dorsal horn. Importantly, SIRT3 deficiency abolished KD's protective effects against neuropathic pain and reduced BHB levels, potentially attributable to diminished expression of hepatic ketone body synthases and spinal ketone body-utilizing enzymes. These findings highlight SIRT3 as a promising therapeutic target for neuropathic pain within the ketogenic diet paradigm, providing a foundation for novel therapeutic strategies.

## Introduction

Neuropathic pain (NPP), a debilitating condition arising from somatosensory nervous system lesions or dysfunction, affects 11-19% of adults globally and constitutes a major therapeutic challenge due to limited efficacy and adverse effects of current treatments 1. Characterized by spontaneous pain, sensory hypersensitivity, and aberrant nociceptive responses, NPP pathophysiology involves neuroinflammatory cascades triggered by nerve injury 2, 3. Pro-inflammatory mediators infiltrate neural tissues, inducing neuroinflammation, nerve hypertrophy, and subsequent mechanical allodynia and thermal hyperalgesia 4. Central to this pathological cascade is the activation and proliferation of spinal microglia in the dorsal horn, which amplify inflammatory signaling and neuronal sensitization 5. Existing treatments for neuropathic pain, a common and multifactorial condition, often inadequately manage this condition and may cause severe side effects 6. The persistent gap between mechanistic insights and clinical management underscores the urgent need for novel therapeutic strategies.

Microglia, the resident immune cells of the central nervous system (CNS), dynamically surveil the neural microenvironment through ramified processes under physiological conditions 7. Following peripheral nerve injury, spinal microglia transition from a surveillance state to an activated phenotype, triggering neuroinflammatory cascades critical to pain pathogenesis 8. Activated microglia produces pro-inflammatory cytokines including tumor necrosis factor-α (TNF-α), interleukin-6 (IL-6), and interleukin-1β (IL-1β), which enhance synaptic transmission in nociceptive circuits and drive central sensitization 9, 10. Concurrently, systemic endocrine mediators such as cortisol, estrogen, and ghrelin are reported to regulate the bidirectional neuroimmune crosstalk and pain processing 11. Dysregulation of these hormone-mediated signaling pathways may facilitate the transition from acute to chronic pain states 11, 12. In addition, mitochondrial dysfunction exacerbates pathological pain via excessive reactive oxygen species (ROS) production. Electron leakage from the electron transport chain generates ROS that promote neuroinflammation by activating spinal microglia and initiating pro-inflammatory signaling cascades. Elevated ROS levels in the spinal cord correlate with both the initiation and persistence of chronic pain, thereby establishing oxidative stress as a key mediator of maladaptive plasticity in pain pathways 13.

The ketogenic diet (KD), a high-fat, low-carbohydrate dietary intervention, has the therapeutic efficacy in epilepsy management by inhibiting neuron inflammation and hyperexcitation through multiple mechanisms 14-16. Its antiepileptic effects arise from metabolic reprogramming that bidirectionally regulates neuroendocrine axis activity and synaptic plasticity, primarily mediated by β-hydroxybutyrate (BHB)-induced histone deacetylase (HDAC) inhibition, which enhances GABAergic neurotransmission to elevate seizure thresholds 17. Neuroprotection is achieved through four synergistic pathways: (1) attenuation of neuronal hyperexcitability via chloride gradient stabilization, (2) potentiation of adenosine-mediated purinergic signaling, (3) metabolic shift from glycolysis to ketolysis, and (4) mitochondrial functional restoration through oxidative stress mitigation 16, 18-20. The diet's anti-inflammatory action involves NADH:NAD⁺ ratio reduction from glucose restriction, suppressing nuclear factor kappa-B (NF-κB)-dependent pro-inflammatory gene expression in immune cells, coupled with triple antioxidant mechanisms-HDAC class I inhibition, nuclear factor erythroid 2-related factor 2 (Nrf2) activation, and NF-κB suppression 21, 22. Experimental models of Alzheimer's disease reveal KD's microglial modulation through nucleotide-binding oligomerization domain-like receptor protein 3 (NLRP3) inflammasome inhibition, reducing IL-1β, caspase-1, and ROS production 23, 24. Notably, the KD may also disrupt the ​​Growth Hormone-Insulin-like Growth Factor 1 (GH-IGF-1) axis by altering ghrelin (GHRP) metabolism, potentially compromising growth and development 25. Despite these characterized immunomodulatory effects in neuroinflammatory and neurodegenerative contexts, the diet's potential role in microglia-mediated neuropathic pain pathogenesis remains unexplored.

Post-translational protein acetylation/deacetylation modifications serve as critical regulatory mechanisms for mitochondrial protein functionality, with sirtuin family members - particularly SIRT3 - exerting principal regulatory effects through NAD^+^-dependent deacetylase activity as demonstrated by studies documenting its NAD^+^-dependent deacetylase activity 26-28. As the most potent mitochondrial deacetylase, SIRT3 has been mechanistically linked to: (1) regulation of mitochondrial permeability transition pore dynamics, (2) modulation of Bcl-2-associated X (Bax)/B-cell lymphoma 2 (Bcl-2) apoptotic signaling ratios, (3) suppression of inflammasome-related proteins including NLRP3 and caspase-1, and (4) attenuation of microglial dysfunction and neuroinflammatory responses 29, 30. Despite these established roles, the potential therapeutic implications of SIRT3 in neuropathic pain management and mitochondrial functional recovery under KD conditions remain insufficiently characterized. This study investigates the analgesic efficacy of KD in neuropathic pain models, mechanistically exploring the dual role of enhanced ketone body metabolism in both pain mitigation and SIRT3-mediated neuroinflammatory regulation. Our results elucidate a novel pathway through which nutritional ketosis exerts neuroprotective effects, revealing critical dependencies on SIRT3 activation for optimal therapeutic outcomes.

## Materials and Methods

### Animals

Male C57BL/6J mice (wild type, n=200; 6-8 weeks, 18-20 g) and *Sirt3*-KO strains (homozygous and heterozygous, n=153 each) were obtained from Naval Medical University and Shanghai Southern Model Biotechnology. Neonatal mice (n=12) provided primary cells. Cohorts were staggered to optimize workflow, with littermate controls prioritized to reduce variability. Mice were housed under specific pathogen-free conditions (22±1°C, 55±5% humidity, 12h light cycles) with ad libitum access to sterilized resources. All experimental protocols were conducted in compliance with the guidelines of the International Association for the Study of Pain and were approved by the Animal Ethics Committee of Shanghai Changzheng Hospital (SYXK[Hu]2022-0011).

### CCI model establishment

Mice were anesthetized with 1% pentobarbital sodium (50 mg/kg, i.p.) for unilateral sciatic nerve exposure. Following surgical preparation of the left hindlimb, a 1 cm nerve-parallel incision exposed the sciatic trunk. Four 1 mm-spaced microconstrictions were applied using calibrated ligation (adjusted to induce transient toe twitching). Layered closure with sterile sutures completed the procedure. Postoperative monitoring continued until predefined humane endpoints.

### Dietary intervention

Control mice received standard chow (16.63% protein, 76.50% carbohydrates; XTM06-005) while KD groups consumed matched isocaloric feed (90% fat; XTKD01). To assess BHB-dependent Sirt3 regulation, *Sirt3^+/-^* and *Sirt3^-/-^* littermates (6-8 weeks) underwent CCI and sham surgery followed by daily oral BHB (150 mg/kg, Sigma #54965 in saline) or vehicle from 24 h post-op until endpoint.

### Serum BHB Level measurement

Submandibular venous blood (200-250 μL) was collected from restrained mice using vacuum-assisted phlebotomy (4.0 mm needle, 30° insertion). Hemostasis was achieved by 2-min compression. Serum was isolated via coagulation (22°C/30 min) and centrifugation (1500 × g, 4°C/15 min) and stored at -80°C (≤2 freeze-thaw cycles). BHB levels were quantified by enzyme-linked immunosorbent assay (ELISA) (Cayman #700190) per manufacturer protocols 31.

### Measurement of mechanical and thermal pain thresholds

Mechanical allodynia was assessed using an electronic von Frey system (EVF-3000, North Coast Medical) during 09:00-14:00 h under double-blind conditions. Mice were acclimated in acrylic chambers with mesh floors (30 min) before testing. A 0.6 mm probe applied ascending forces (0-15 g) to the left hindpaw plantar surface, with five trials at 3-min intervals to prevent sensitization.

A Hargreaves apparatus (IITC Model 390G) measured paw withdrawal latency using a 50 W infrared beam (5×8 mm spot) directed at the left hindpaw. Mice acclimated in temperature-controlled enclosures (25±1°C) were tested with ≥10-min inter-stimulus intervals. Trials capped at 20 s (tissue protection) and ≤3 valid trials/session, excluding non-specific behaviors.

### Measure of weight, blood ketone, and blood glucose

Weekly body mass (±0.1 g precision) was recorded at 08:00-09:00 h. Fasted blood samples (3-5 μL) from tail veins were analyzed using: FreeStyle Optium™ Neo H analyzer (Abbott Laboratories; NMPA 20152221016), Glucose dehydrogenase-based strips (GDH; NMPA 20192402119) and β-HB electrochemical strips (NMPA 20172400719).

### BV-2 cell and primary microglia culture

The BV-2 microglial cell line (Cat# CC-0301, Quicell, Shanghai) was maintained in Dulbecco's Modified Eagle Medium (DMEM) at 37°C/5% CO₂ with routine subculture at 80% confluence. Lipopolysaccharide (LPS, 1 μg/mL; Sigma #L2880), Decanoic acid (60 μM; Cayman #14403), R-3-hydroxybutyric acid (1.5 mM; Sigma #H6501) and Low-glucose DMEM (1.0 g/L D-glucose) to stimulate cells.

Neonatal mice (n=12) were anesthetized and euthanized for brain collection. Disinfected brains were minced (500 μm grids) and digested with 0.125% trypsin-EDTA (Gibco #25200072, 37°C/15 min), neutralized using DMEM/10% FBS (Gibco #10437028). Cells were centrifuged (300 × g, 10 min, 4°C), cultured in complete medium (37°C, 5% CO₂) with medium changes every 24 h, followed by 14-day culture with thermostatic shaking (180 rpm, 37°C). *Sirt3* genotypes were confirmed by PCR (Vazyme PD111, primers in Table S1).

### Western blotting

Tissues/cells were homogenized in RIPA lysis buffer (Epizyme #PC101) with protease/phosphatase inhibitors (Epizyme #GRF103) using bead-mill homogenization (3×30s cycles, 25 Hz, 4°C). Lysates were cleared by centrifugation (12,000×g, 15 min, 4°C) and quantified via bicinchoninic acid assay (BCA) assay (Epizyme #ZJ102). Proteins (30-50μg) were resolved on gradient Bis-Tris gels and transferred to PVDF membranes (400mA, 30-120 min). After blocking (Epizyme #PS108P), membranes were probed with: BDH1 (1:5000, Proteintech #15417-1-AP); OXCT1 (1:5000, #12175-1-AP); UCP2 (1:1000, #11081-1-AP); PGC-1α (1:10000, #66369-1-Ig); PPARγ (1:20000, #66936-1-IG); SIRT3 (1:1000, Abcam #ab217319); βIII-Tubulin (1:1000, #ab18207); GAPDH (1:10000, #ab8245); SIRT3 (1:1000, #ab217319); NF-κB p65 (1:1,000; CST#8242); Phospho-p65 (1:1,000; #3033). Species-matched HRP-secondary antibodies (CST, anti-mouse #7076S, anti-rabbit #7074S, 1:2000, 1 h, 25°C) were used. Signals were acquired on ChemiDoc MP (Bio-Rad) and quantified in ImageJ.

### qRT-PCR

Total RNA was extracted from murine spinal cord tissues and microglia cultures using Monzol™ Reagent Pro (Monad Biotech, #MI20201S), with RNA purity verified by A260/A280 ratios (1.8-2.0). cDNA was synthesized from 1 μg RNA using HiScript II Reverse Transcriptase (Vazyme, #R211), followed by quantitative PCR (qPCR) performed with ChamQ SYBR Master Mix (Vazyme, #Q711) on a QuantStudio 5 system (Applied Biosystems). The thermal cycling protocol consisted of an initial denaturation at 95°C for 30 seconds, followed by 40 cycles of 95°C for 10 seconds and 60°C for 30 seconds. Validated primers (Sangon Biotech, Table S2) were used in triplicate reactions. Relative gene expression was calculated using the 2-ΔΔCt method, normalized to β-actin or GAPDH.

### RNA sequencing and analysis

Initial processing involved adapter trimming (Skewer, Phred score <20) and quality control (FastQC v0.11.2). Clean reads were then aligned to the mm10/GRCm38 reference genome (GENCODE M21 annotations) using STAR v2.7.10a in strand-specific mode. Transcript assembly was performed using StringTie v2.1.4. Differentially expressed genes (DEGs) were identified using the R packages DESeq2 and visualized with ggplot2, with thresholds set at P<0.05 and |log₂FC| ≥ 1. Subsequently, functional annotation was conducted using Gene Ontology (GO) and Kyoto Encyclopedia of Genes and Genomes (KEGG) analyses with the clusterProfiler v4.0 package. Additionally, gene co-expression networks were constructed based on Spearman's rank correlation coefficient, visualized using Cytoscape, and statistical significance was determined with a threshold of P < 0.05.

### Immunofluorescence

Tissue sections underwent microwave antigen retrieval (EDTA, 10 mM, pH 8.0, 95°C, 20 min), blocked with 5% bovine serum albumin containing 0.1% Triton X-100, and incubated with primary antibodies at 4°C overnight: Iba1 (1:200, CST #17198), GFAP (1:800, CST #3670), BDH1 (1:500, Proteintech 15417), OXCT1 (1:1000, 12175), SIRT3 (1:500, Abcam ab217319), HMGCS2 (1:100, ab309164) and 8-OHdG (1:1000, ab62623). After PBS washes, sections were stained with Alexa Fluor secondaries (1:500, 1 h) and DAPI (0.5 μg/mL). Fluorescent images were acquired using a laser-scanning confocal microscope (Nikon A1R) with consistent exposure settings across compared samples.

### Transmission electron microscopy

Mice were anesthetized (pentobarbital 50 mg/kg, i.p.) and transcardially perfused with ice-cold 0.9% NaCl. Spinal dorsal horn tissue samples (1 mm³) were fixed in a solution containing 2.5% glutaraldehyde and 0.1 M phosphate buffer (pH 7.4, 4°C for 4 hours), followed by post-fixation in 1% osmium tetroxide (4°C for 2 hours), then dehydrated through graded ethanol and embedded in Epon 812. Ultrathin sections (60 nm, Leica UC7) were stained with uranyl acetate/lead citrate and imaged using Hitachi HT7700 TEM.

### Mitochondrial isolation

Mitochondria were isolated from spinal cord tissue using the Mitochondrial Extraction Kit (Beyotime Biotechnology, #C3606, Shanghai, China) following the manufacturer's protocol 32. Tissues were homogenized in ice-cold extraction buffer supplemented with protease inhibitors (1 mM PMSF, 1× EDTA-free protease inhibitor cocktail) using a Dounce homogenizer (20 strokes). Sequential centrifugations were performed at 1,000 × g (10 min, 4°C) to remove cellular debris, followed by 12,000 × g (30 min, 4°C) to pellet intact mitochondria.

### JC-1 staining

Cells were cultured in 6-well plates under standard conditions (37°C, 5% CO₂) and stained with JC-1 (5 μM, Beyotime #C2003S) for 30 min. ΔΨm was analyzed via: (1) Confocal imaging (BioTek Lionheart FX, 60×) capturing Z-stacks (5 slices, 0.5 μm intervals) with dual-channel detection (aggregates: 585/590-650 nm; monomers: 485/510-550 nm); (2) Flow cytometry (BioTek Synergy HTX) quantifying ≥10,000 events/sample (FITC/PE channels), analyzed via FlowJo v10.8.1. For isolated spinal mitochondria (12,000 × g/30 min), JC-1 (10 μg/mL) fluorescence was quantified in MAS buffer using a Tecan M200 Pro microplate reader (530/590 nm).

### Mitochondrial DNA copy numbers

Genomic DNA was extracted from treated cells and tissues using DNeasy Kit (Qiagen #69504) and quantified (A260/A280 ratio 1.8-2.0). mtDNA/ncDNA ratios were determined by quantitative PCR (SYBR Green Master Mix, Takara #RR420A) on a Bio-Rad CFX96 system with primers (Table S2) 33, 34. Cycling conditions: 95°C for 3 min, followed by 40 cycles of 95°C for 10 s and 60°C for 30 s. Amplification efficiency (90-105%) was validated by melt curve analysis. Data were analyzed via ΔΔCt method.

### Oxygen Consumption Rate (OCR) measurement

BV-2 microglia in 96-well flat-bottom black-walled plates (Corning #3904) were analyzed using BBoxiProbe™ R01 Oxygen Sensor (BioBox #BB-48311). Post-treatment, oxygen-sensitive nanoprobe (Pt-porphyrin, 10 μL per well) was added and stabilized with hypoxia-compatible agarose sealant (100 μL 0.5% in PBS). Kinetic fluorescence measurements (380/650 nm) were recorded every 5 min for 120 h using a FLUOstar Omega reader (37°C). Data normalized to Hoechst 33342-quantified cell counts (1 μg/mL, 10 min) 35.

### Extracellular Acidification Rate (ECAR) analysis

BV-2 cells in 96-well plates were assessed using ECAR Pro™ Assay (BioBox #BB-48501). Following 12 h treatment and 2 h CO_2_-free incubation (37°C) for pH stabilization, pH-sensitive probe (Ex/Em = 488/580 nm) was loaded in bicarbonate-free DMEM (Gibco #12800-017). Real-time fluorescence was monitored every 5 min for 120 min (FLUOstar Omega, 37°C). Acidification rates from linear-phase measurements (20-80 min) were normalized to BCA-quantified protein (Pierce #23225) 36.

### ATP evaluation

ATP levels were measured using a luciferase-based assay (Beyotime #S0026). Samples homogenized in ice-cold lysis buffer (100 μL/10 mg tissue or 10⁶ cells) were centrifuged (12,000 × g, 10 min, 4°C), and supernatants analyzed in white 96-well plates (Corning #3912) with FLUOstar Omega (1-s integration). Parallel BCA assays (Pierce #23225, 562 nm) quantified protein content for normalization (nmol ATP/mg protein).

### ROS detection in BV2 microglia and spinal cord tissues

BV2 microglial cells were seeded onto glass-bottom chamber slides, washed with PBS, and incubated with 10 μM DCFH-DA (Beyotime #S0033S) in serum-free DMEM (37°C, 30 min, dark). After three PBS washes, live-cell imaging was performed using a Leica TCS SP8 confocal microscope (40×) with Z-stacks (1 μm intervals) at Ex/Em 488/525nm under standardized parameters (15% laser power, 600 V gain, 500 ms exposure).

For spinal cord sections (10 μm), autofluorescence was quenched with TrueBlack® (Biotium #23007, 5 min), followed by ROS staining with 5 μM CellROX® Deep Red (Thermo Fisher #C10422, 37°C for 30 min, dark) and DAPI counterstaining (1 μg/mL). Sections were mounted with ProLong™ Diamond (#P36970) and imaged on a Zeiss LSM 980 system.

### Data analysis

Statistical analyses were conducted using GraphPad Prism 9.0 and R 4.3.1, with continuous variables expressed as mean ± standard deviation (SD) derived from three or more independent biological replicates. Intergroup comparisons were performed as follows: unpaired Student's t-tests with Welch's correction for two-group comparisons and one-way ANOVA with Tukey's post hoc test for multi-group analyses, with statistical significance thresholds defined as *P < 0.05, **P < 0.01, and ***P < 0.001. Western blot bands (ImageJ) were quantified with loading controls normalization and averaged from triplicate technical replicates.

## Results

### KD alleviated central neural inflammation and neuropathic pain

**Figure 1:**
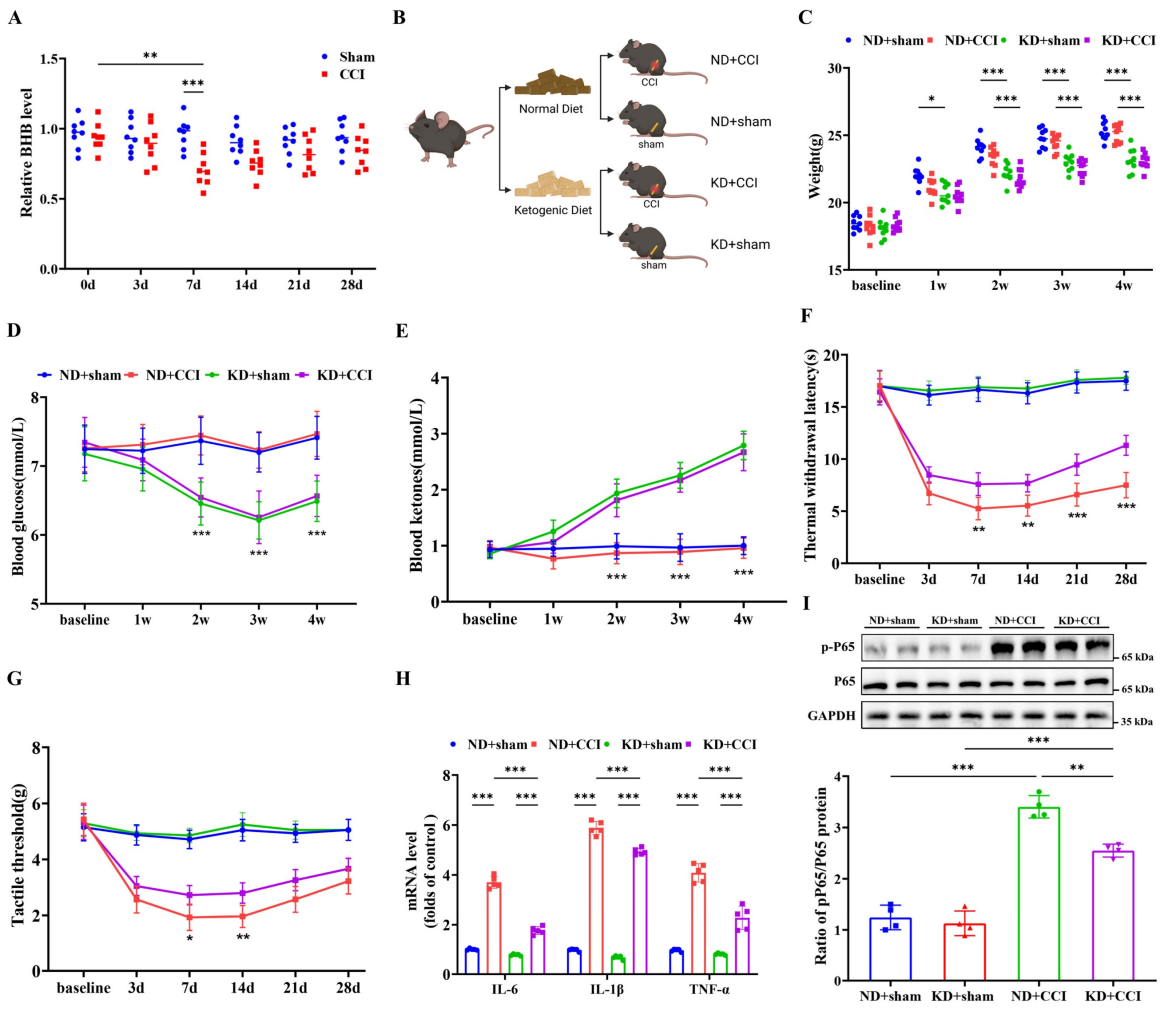
The ketogenic diet alleviates central nervous system inflammation and neuropathic pain. (A) Serum BHB levels pre-modeling and at 3-, 7-, 14-, 21-, and 28-day post-modeling time points (n = 8). (B) Schematic of the experimental design. (C) Body weight trajectories (n = 9). (D) Blood glucose levels (n = 9). (E) Circulating ketone body levels (n = 9). (F) Thermal nociceptive stimulus responses in mice (n = 9). (G) Mechanical nociceptive stimulus responses in mice (n = 9). (H) mRNA levels of pro-inflammatory cytokines (TNF-α, IL-1β, and IL-6) in the spinal cord at day 7 post-CCI (n = 4). (I) Protein expression of phosphorylated NF-κB p65 (p-p65) with GAPDH as a loading control (n = 4). *p < 0.05, **p < 0.01, ***p < 0.001.

To investigate the involvement of BHB biosynthesis in neuropathic pain development, serum BHB levels were quantified in both CCI mice and sham-operated controls. CCI modeling induced significant reductions in serum BHB concentrations, with the most pronounced reduction observed 7 days post-surgery compared to sham controls (Figure 1A). To assess the neuroprotective potential of KD, both sham and CCI groups received KD administration starting on the day of CCI induction (Figure 1B). Systematic assessment revealed that KD implementation caused three distinct metabolic alterations: progressive reductions in body weight, sustained hypoglycemia, and elevated circulating ketone bodies. These changes became statistically significant from day 7 onward and persisted until experimental termination at day 28 (Figures 1C-1E). Behavioral validation confirmed successful neuropathic pain model establishment, as demonstrated by progressive thermal hyperalgesia and mechanical allodynia (Figures 1F and 1G). KD intervention significantly ameliorated both nociceptive thresholds starting at day 7, with sustained therapeutic effects observed until day 28. Based on the peak treatment effect observed 7 days post-CCI, this time point was selected as the observation window for the subsequent main study. Histological evaluation demonstrated KD-mediated suppression of pro-inflammatory cytokines (IL-6, IL-1β, TNF-α) and phosphorylated NF-κB p65 (p-p65) in the spinal dorsal horn of CCI mice (Figures 1H and 1I). These results collectively demonstrate that KD attenuates central neuroinflammation and neuropathic pain progression.

### KD inhibited microglia activation and attenuated mitochondrial damage *in vivo*

**Figure 2:**
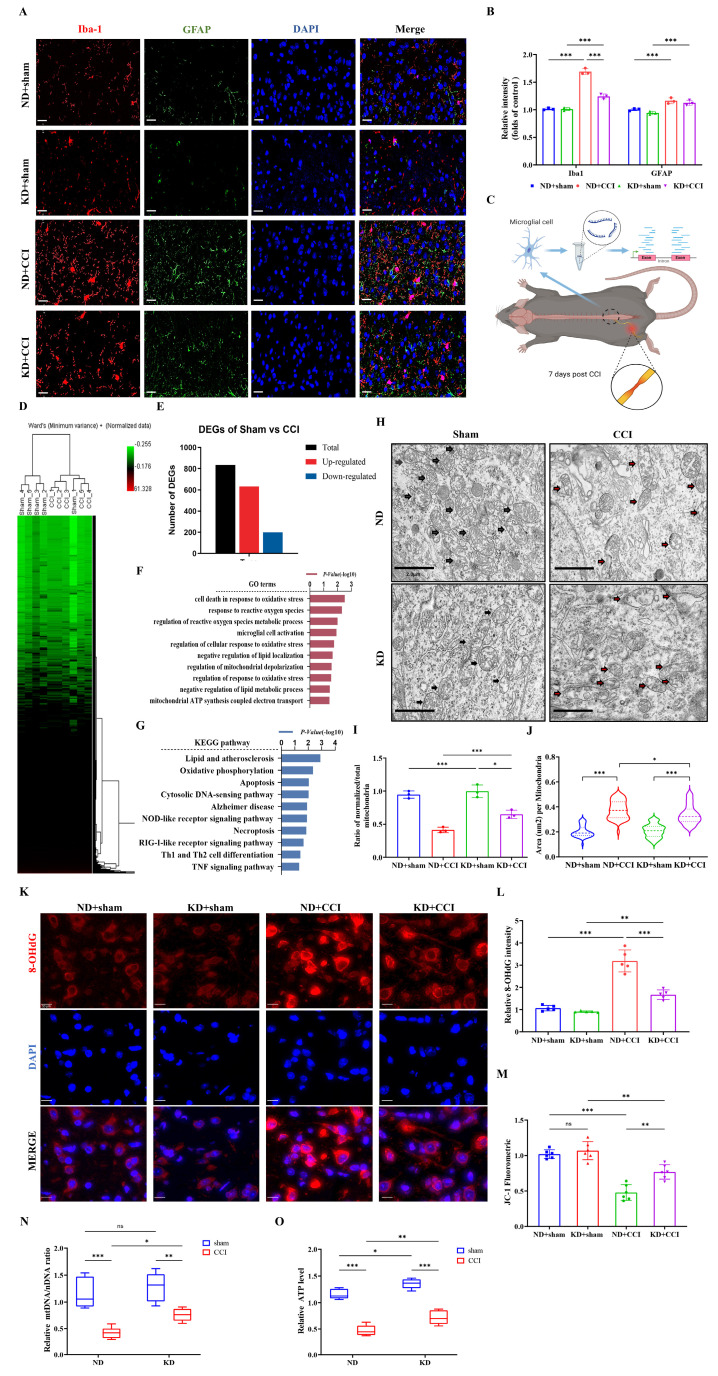
The ketogenic diet modulates microglial activation and mitochondrial homeostasis in CCI-induced neuropathic pain. (A) Representative immunofluorescence images of spinal Iba-1⁺ microglia (red) and GFAP⁺ astrocytes (green) at 7 days post-CCI, counterstained with DAPI (blue). Scale bars: 25 µm. (B) Quantification of Iba-1 and GFAP fluorescence intensity (n = 3). (C) Experimental Workflow of GSE180627 Data Analysis. (D) HCA of microglial transcriptomes (from GSE180627) (n = 5). (E) Counts of DEGs. (F, G) GO and KEGG pathway enrichment analysis of CCI versus Sham microglia. (H) Transmission electron micrographs of spinal dorsal horn mitochondria (black arrows: intact cristae; red arrows: swollen or damaged cristae). Scale bars: 2 µm. (I) Normalized-to-Total Mitochondrial Ratio (n=3). (J) Area of the mitochondria. n=50 mitochondria / group. From top to bottom, the dashed lines in the violin plots represent upper quartile, median, and lower quartile. (K) 8-OHdG⁺ oxidative damage foci (red). Scale bars: 10 µm. (L) 8-OHdG fluorescence intensity (n = 5). (M) JC-1 mitochondrial membrane potential (n = 6). (N, O) Mitochondrial DNA copy number and ATP levels (n = 5). Data are presented as mean ± SEM. *p < 0.05, **p < 0.01, ***p < 0.001.

To explore the cellular mechanisms underlying the beneficial effects of the KD, we examined microglial activation in the spinal dorsal horn. KD significantly inhibited the CCI-induced reduction of Iba-1 expression (a microglial activation marker), but had no significant effect on GFAP (an astrocyte activation marker) (Figures 2A and 2B). Subsequent reanalysis of the GSE180627 dataset (available in the GEO database), which profiles microglial gene expression in the ipsilateral lumbar spinal cord at day 7 post-CCI across sexes (Figure 2C and 2D) and identification of differentially expressed genes (DEGs) (Figure 2E). Gene Ontology (GO) analysis identified alterations in microglial processes related to oxidative stress, mitochondrial regulation, and immune modulation (Figure 2F), while KEGG pathway analysis confirmed dysregulation of programmed cell death (apoptosis/necroptosis) and neuroinflammatory signaling (NOD-like/TNF pathways) (Figure 2G). Quantitative evaluation of both the normalized-to-total mitochondrial ratio and cross-sectional mitochondrial area revealed that CCI induced significant mitochondrial ultrastructural damage in spinal dorsal horn tissues across experimental groups, characterized by matrix vacuolization, cristae fragmentation, and membrane disruption. KD intervention significantly attenuated these pathological ultrastructural alterations (Figure 2H-2J). We further assessed oxidative stress biomarkers (8-hydroxy-2'-deoxyguanosine, 8-OHdG) and mitochondrial membrane potential (ΔΨm) in spinal cord tissues. As shown in Figures 2K and 2L, CCI induced a significant elevation in 8-OHdG levels (indicative of oxidative stress), accompanied by a decrease in mitochondrial membrane potential (Figure 2M). Additionally, mitochondrial DNA (mtDNA) copy number decreased by over 50% in CCI mice (Figure 2N), with a corresponding reduction in adenosine triphosphate (ATP) levels in the spinal dorsal horn (Figure 2O). Notably, KD attenuated oxidative damage (evidenced by reduced 8-OHdG levels) and enhanced mitochondrial membrane potential and mtDNA copy number, resulting in increased ATP production (Figure 2O). These findings collectively suggest that KD exerts neuroprotective effects through mitigation of oxidative stress and enhancement of mitochondrial function in neuropathic pain.

### BHB inhibited inflammatory responses and ameliorated mitochondrial damage in microglia *in vitro*

**Figure 3:**
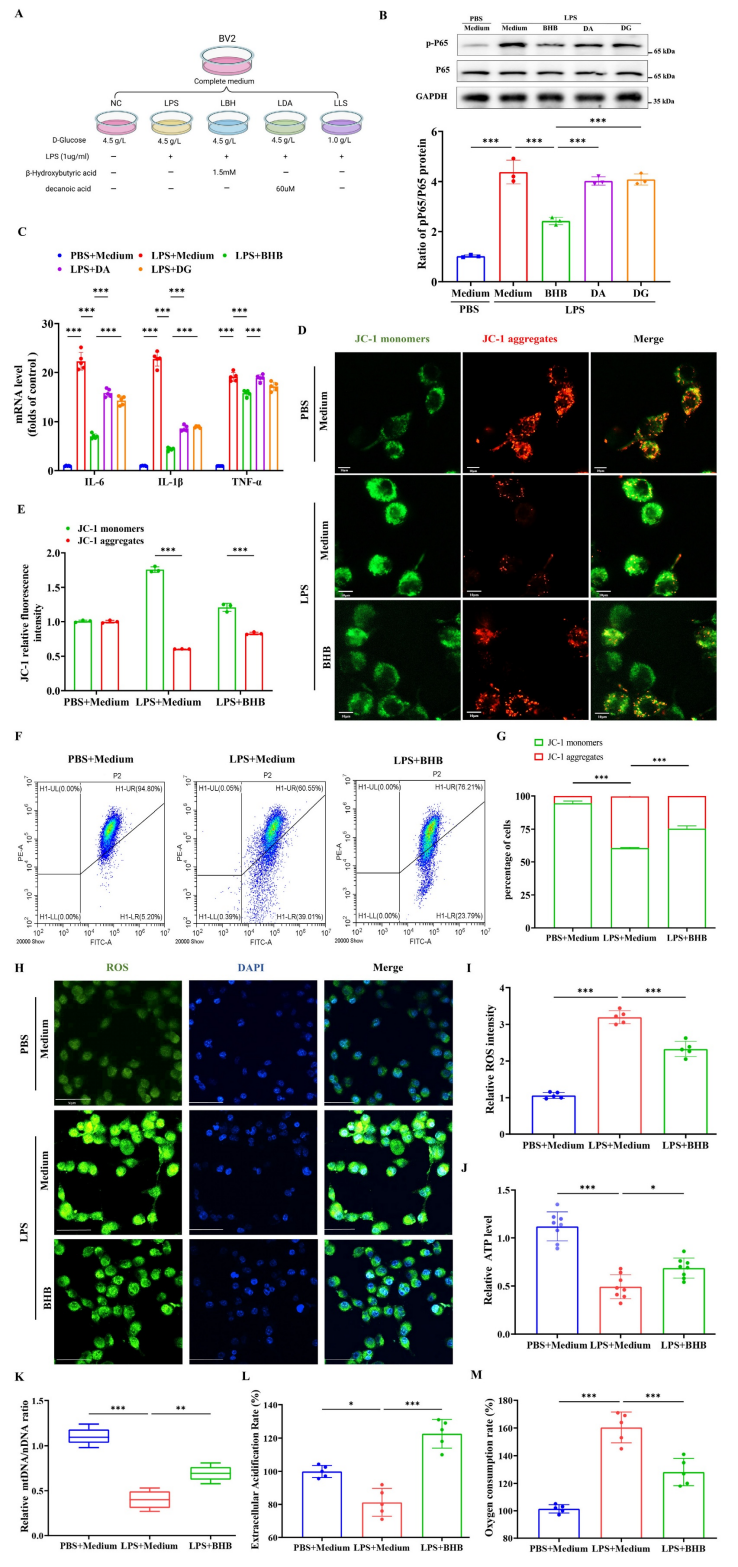
BHB attenuates LPS-induced microglial inflammation and mitochondrial dysfunction. (A) Schematic of the experimental design. (B) Protein expression of phosphorylated NF-κB p65 (p-p65) in LPS-stimulated BV2 microglia treated with β-hydroxybutyrate (BHB group), decanoic acid (LDA group), or low glucose (LLS group), with GAPDH as a loading control (n = 3). (C) mRNA levels of pro-inflammatory cytokines (TNF-α, IL-1β, and IL-6) measured by qRT-PCR (n = 4). (D) JC-1 mitochondrial membrane potential staining (red: J-aggregates [polarized]; green: monomers [depolarized]). Scale bars: 10 µm. (E) Quantification of the JC-1 fluorescence ratio (n = 3). (F, G) Flow cytometric analysis of mitochondrial polarization (n = 4). (H) ROS fluorescence imaging. Scale bars: 50 µm. (I) Quantification of ROS intensity (n = 5). (J) Cellular ATP levels (n = 8). (K) Mitochondrial DNA copy number (n = 5). (L) and OCR (M) detection in three groups of cells (n = 5). Data are presented as mean ± SEM. *p < 0.05, **p < 0.01, ***p < 0.001.

To identify the specific components of the KD responsible for its anti-inflammatory effects, BV-2 microglia were treated with three KD components: β-hydroxybutyrate (BHB group), fatty acids (LDA group), and low glucose (LLS group) (Figure 3A). BHB treatment specifically suppressed LPS-induced NF-κB activation (phosphorylation of p65), whereas the LDA and LLS groups showed no significant effects (Figure 3B), identifying BHB as the critical anti-inflammatory component. Quantitative PCR further confirmed BHB-mediated downregulation of pro-inflammatory cytokines (IL-6, IL-1β, TNF-α) at the mRNA level (Figure 3C). Mitochondrial functional assays revealed that BHB treatment preserved mitochondrial membrane potential in LPS-challenged BV-2 cells (Figures 3D and 3E), with flow cytometry validating reduced mitochondrial depolarization (Figures 3F and 3G). ROS quantification demonstrated that BHB effectively attenuated LPS-induced oxidative stress (Figures 3H and 3I). Metabolic profiling indicated that BHB reversed LPS-mediated ATP depletion (Figure 3J) and restored mtDNA copy numbers (Figure 3K). We further observed that BHB restored LPS-disrupted ECAR to baseline (Figure 3L) and normalized OCR (Figure 3M). These data collectively establish that BHB counteracts LPS-induced mitochondrial dysfunction and oxidative stress in microglia, suggesting its pivotal role in KD-mediated neuroprotection via dual modulation of inflammatory signaling and metabolic homeostasis.

### BHB alleviated central neural inflammation and neuropathic pain by upregulating SIRT3 expression

**Figure 4:**
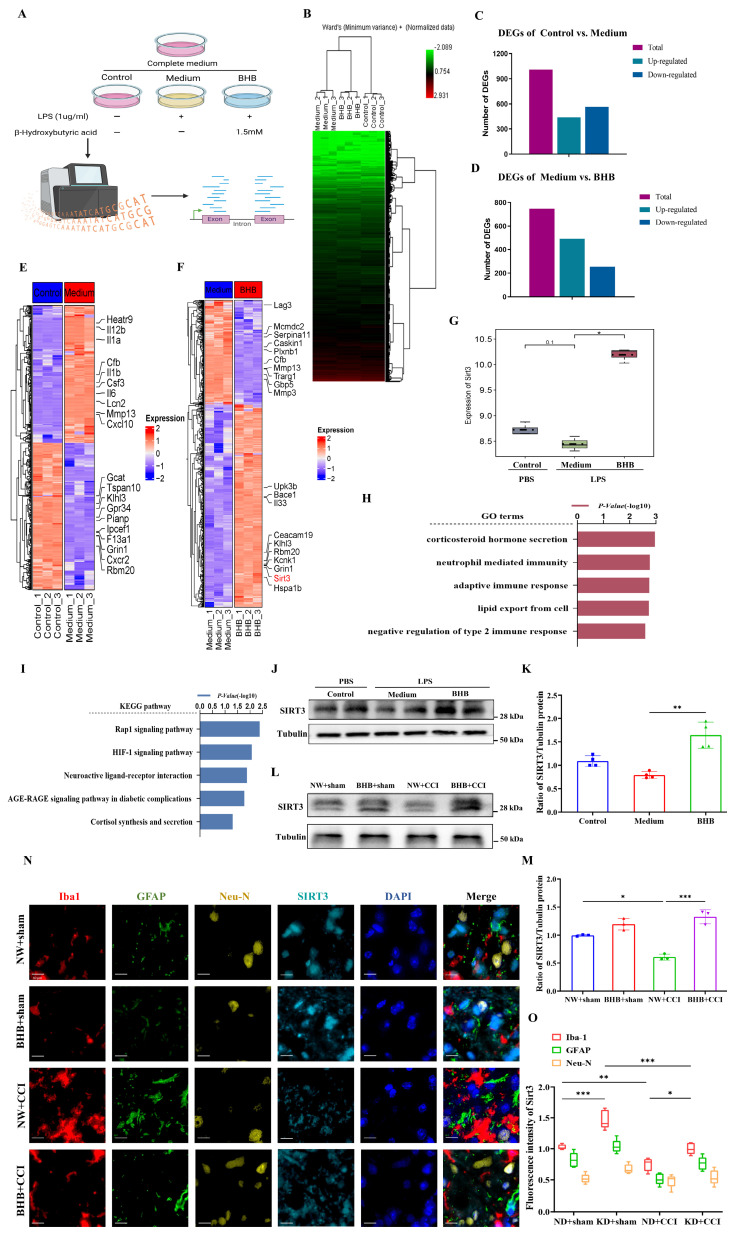
BHB inhibits the inflammatory response by upregulating SIRT3 expression. (A) Schematic of sample sources and experimental groups. (B) HCA of BV2 cells (n = 3). (C, D) Bar graphs showing the number of DEGs. (E) Heatmap of significantly up- and down-regulated genes in the control group versus medium group. (F) Heatmap of significantly up- and down-regulated genes in the medium group versus BHB group. (G) Expression of SIRT3 in the three groups (n = 3). (H) GO analysis results. (I) KEGG pathway enrichment results. (J) SIRT3 protein expression in BV2 cells upon LPS stimulation with or without BHB. Tubulin was used as a loading control. (K) SIRT3 protein expression in BV2 cells (n = 4). (L) Expression of SIRT3 protein in the spinal dorsal horn at day 7 post-CCI modeling. Mice received regular and quantitative administrations of normal saline or BHB following anesthetic recovery on the first day of modeling. Tubulin was used as a loading control. (M) SIRT3 protein expression in the spinal cord at day 7 post-CCI modeling (n = 4). (N) Immunofluorescence staining results for Iba-1 (red), GFAP (green), NeuN (yellow), and SIRT3 (cyan) in the spinal cord at day 7 post-CCI modeling, counterstained with DAPI (blue). Scale bars: 10 µm. (O) Fluorescence intensity of Iba-1, GFAP, and NeuN co-localized with SIRT3. Data are shown as mean ± SEM. *p < 0.05, **p < 0.01, ***p < 0.001.

Systematic experimentation established BHB as the principal KD component suppressing microglial inflammatory activation. To delineate the molecular basis of KD-mediated neuropathic pain relief, we conducted mRNA sequencing on LPS-stimulated BV-2 microglia treated with phosphate-buffered saline (PBS) or BHB (Figures 4A and 4B). LPS challenge induced substantial transcriptomic reprogramming, with BHB treatment eliciting 747 differentially expressed genes (493 upregulated, 254 downregulated) (Figures 4C and 4D). Functional annotation revealed that BHB-modulated genes were enriched in immune processes (Gene Ontology [GO] analysis, Figure 4H) and oxidative stress/metabolic pathways (Kyoto Encyclopedia of Genes and Genomes [KEGG] analysis, Figure 4I). Notably, BHB upregulated SIRT3 expression (Figures 4F and 4G), prompting focused investigation of this mitochondrial deacetylase. Quantitative immunoblotting confirmed LPS-induced SIRT3 suppression in both cellular models (Figures 4J and 4K) and spinal cord tissues (Figures 4L and 4M), and this suppression was reversed by BHB treatment. Cell-type-specific co-localization analysis demonstrated predominant SIRT3 expression changes in Iba-1⁺ microglia compared to GFAP⁺ astrocytes or NeuN⁺ neurons following CCI and KD interventions (Figures 4N and 4O). These findings identify SIRT3 as a critical regulator of microglial-mediated oxidative stress and inflammatory responses, establishing a mechanistic link between BHB-dependent effects and the therapeutic efficacy of the KD in neuropathic pain.

### *Sirt3* deficiency abolishes the anti-inflammatory effects and alleviation neuropathic pain of BHB

**Figure 5:**
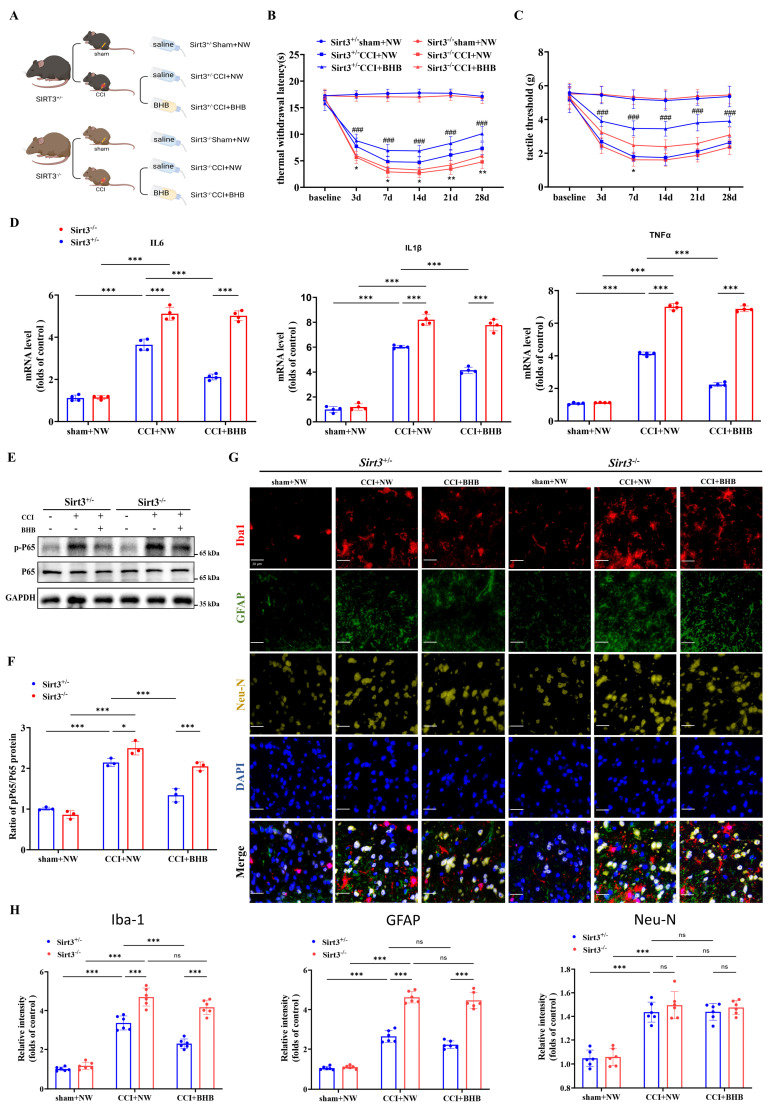
* Sirt3* deficiency abolishes BHB-mediated neuroprotection in CCI-induced neuropathic pain. (A) Schematic of the experimental design. (B, C) Thermal withdrawal latency (B) and mechanical threshold (C) trajectories in *Sirt3^+/-^* and *Sirt3^-/-^* mice before surgery and at 3, 7, 14, 21, and 28 days post-CCI (n = 5). Statistical comparisons: *Sirt3^-/-^*CCI+NW vs. *Sirt3^+/-^* CCI+NW; #*Sirt3^+/-^*CCI+BHB vs. *Sirt3^-/-^*CCI+BHB (*/#p<0.05, **/##p<0.01, ***/###p<0.001). (D) mRNA levels of spinal pro-inflammatory cytokines (TNF-α, IL-1β, and IL-6) at day 7 post-CCI (n = 4). (E, F) Protein expression of phosphorylated NF-κB p65 (p-p65) with GAPDH as a loading control (n = 4). (G) Triple immunofluorescence staining of spinal Iba-1⁺ microglia (red), GFAP⁺ astrocytes (green), and NeuN⁺ neurons (yellow), counterstained with DAPI (blue). Scale bars: 20 µm. (H) Quantification of fluorescence intensity for microglial, astrocytic, and neuronal markers. Data are shown as mean ± SEM. *p < 0.05, **p < 0.01, ***p < 0.001.

To further confirm the role of SIRT3 in BHB-mediated reduction of CNS inflammation and neuropathic pain, we established CCI models in *Sirt3^+/-^* and *Sirt3^-/-^* mice, followed by oral BHB administration (Figure 5A; Figure S1). *Sirt3* deficiency completely abrogated BHB's analgesic effects on thermal hyperalgesia and mechanical allodynia thresholds (Figures 5B and 5C) and abolished BHB-induced suppression of spinal pro-inflammatory cytokines (IL-6, IL-1β, TNF-α) and phosphorylated NF-κB p65 (p-p65) (Figures 5D-5F). Immunofluorescence quantification across six experimental groups revealed that CCI upregulated glial and neuronal markers (Iba-1, GFAP, NeuN) independently of SIRT3 status, whereas BHB administration significantly downregulated all three markers. Notably, SIRT3 deficiency selectively reversed BHB's effects, with the maximal impact observed on microglial Iba-1 expression (Figures 5G and 5H). These data collectively indicate that SIRT3 is essential for BHB-driven regulation of neuroinflammation and glial activation.

### *Sirt3* deficiency abolished the protective effects of BHB on neuropathic pain by impairing mitochondrial function

**Figure 6:**
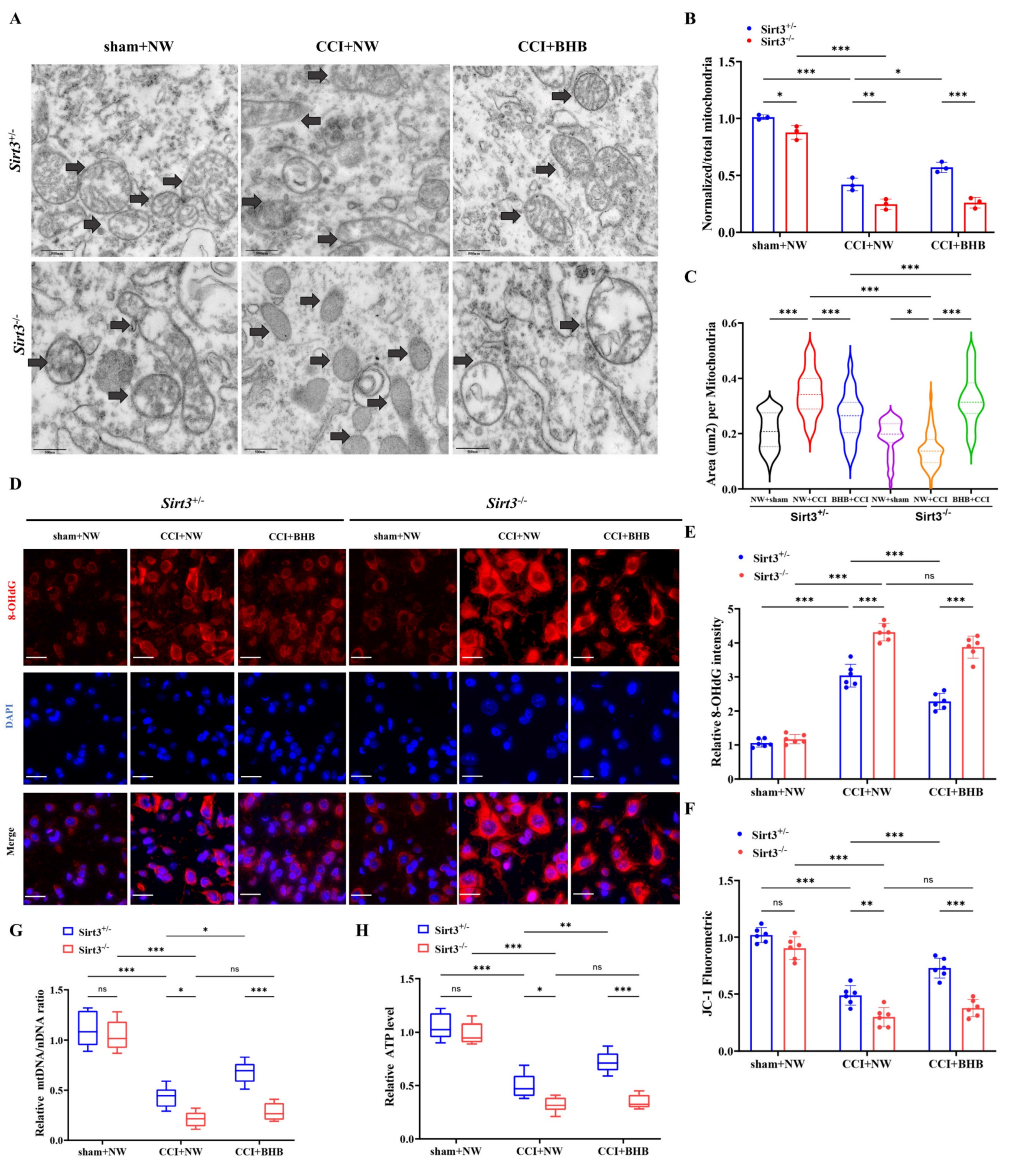
* Sirt3* ablation abrogates BHB-mediated mitochondrial protection and oxidative stress mitigation (A) Transmission electron micrographs of spinal dorsal horn mitochondria across experimental groups. Black arrows: mitochondria. Scale bars: 500 nm (n=3). (B) Normalized-to-Total Mitochondrial Ratio (n=3). (C) Area of the mitochondria. n=50 mitochondria / group. From top to bottom, the dashed lines in the violin plots represent upper quartile, median, and lower quartile. (D) 8-OHdG⁺ oxidative damage foci (red). Scale bars: 10 µm. (E) 8-OHdG fluorescence quantification (n=5). (F) JC-1 mitochondrial membrane potential flow cytometry results (n=6). (G) mtDNA copy number (n=5). (H) ATP production levels (n=5). Data: mean ± SEM. *p<0.05, **p<0.01, ***p<0.001.

Ultrastructural analysis using transmission electron microscopy revealed that BHB ameliorated CCI-induced mitochondrial pathologies, including cristae rupture and swelling, in *Sirt3^+/-^* mice, but exhibited no therapeutic efficacy in *Sirt3^-/-^* counterparts (Figures 6A-6C). Genetic ablation of SIRT3 exacerbated mitochondrial damage, manifested by membrane disintegration and matrix leakage irrespective of BHB administration. Quantitative assessment of oxidative stress markers demonstrated that *Sirt3^-/-^* mice exhibited elevated 8-OHdG expression following CCI, an effect attenuated by BHB in Sirt3^+/-^ mice but not in *Sirt3^-/-^* genotypes (Figures 6D and 6E). Consistent with these findings,* Sirt3* knockout nullified BHB's protective effects on mitochondrial bioenergetics, as evidenced by impaired membrane potential restoration (Figure 6F), failure to reverse mtDNA depletion (Figure 6G), and persistent ATP synthesis deficits (Figure 6H). Similar trends were observed for ROS levels in spinal cord tissue (Figure S2). These data collectively establish SIRT3 as an indispensable mediator of BHB-mediated mitochondrial stabilization and antioxidant effects in neuropathic pain pathogenesis, underscoring its critical role in mitochondrial functional preservation and oxidative damage mitigation.

### *Sirt3* deficiency abolished the increased the expression of UCP2 and PGC-1α by BHB in the dorsal horn of the spinal cord

**Figure 7:**
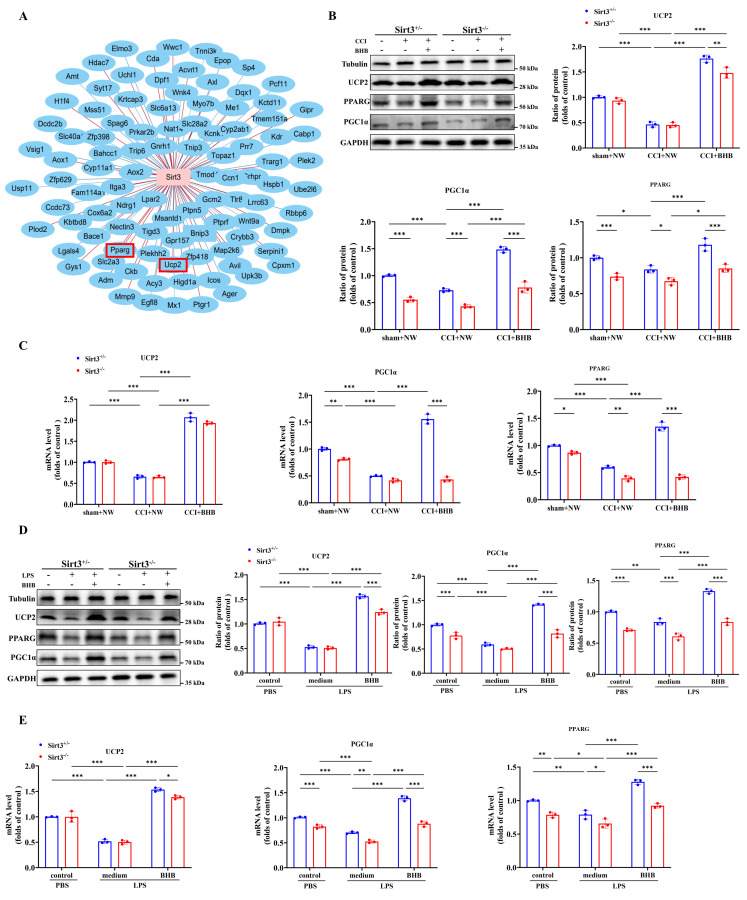
*Sirt3* deficiency abolishes BHB-induced upregulation of UCP2 and PGC-1α in the spinal dorsal horn. (A) Hierarchical clustering of mitochondria-associated DEGs across treatment groups. (B) Protein expression of UCP2, PPARγ, and PGC-1α in the spinal cord of *Sirt3^+/-^* and *Sirt3^-/-^* mice at day 7 post-CCI with BHB or saline administration (Tubulin/GAPDH loading controls; n = 3). (C) Corresponding mRNA levels (n = 3). (D) Protein expression of UCP2, PPARγ, and PGC-1α in primary microglia under LPS stimulation with or without BHB (Tubulin/GAPDH loading controls; n = 3). (E) Quantification of microglial mRNA levels (n = 3). Data are shown as mean ± SEM. *p < 0.05, **p < 0.01, ***p < 0.001.

Gene co-expression analysis revealed a strong correlation between SIRT3 and mitochondrial function-associated genes under BHB intervention, with particularly significant associations observed among peroxisome proliferator-activated receptor gamma (Pparg), uncoupling protein 2 (Ucp2), and SIRT3 (Figure 7A). PGC-1α (peroxisome proliferator-activated receptor gamma coactivator 1 alpha), a Pparg coactivator, modulates energy metabolism-related biological processes 37. Building on established knowledge of the UCP2-SIRT3-PGC-1α axis in mitochondrial quality control and oxidative stress mitigation 38, we investigated its unexplored role in neuropathic pain. Systematic evaluation of Ucp2, Pparg, and PGC-1α expression across *in vivo* and *in vitro* models demonstrated that CCI significantly suppressed both mRNA and protein levels of these targets in *Sirt3^+/-^* and *Sirt3^-/-^* mice (Figures 7B and 7C). BHB administration restored CCI-induced transcriptional and translational deficits in a *Sirt3*-dependent manner, with complete abolition of this rescue effect in *Sirt3-*deficient spinal tissues. In LPS-challenged primary microglia, *Sirt3* deficiency exacerbated the LPS-driven downregulation of Ucp2, Pparg, and PGC-1α. Conversely, BHB intervention attenuated the LPS-induced reductions in mRNA and protein expression of Ucp2, Pparg, and PGC-1α, an effect abolished by *Sirt3* deficiency in primary microglia (Figures 7D and 7E). These results mechanistically link BHB's anti-neuroinflammatory effects to SIRT3-mediated activation of the UCP2-PGC-1α regulatory axis, providing novel insights into mitochondrial pathway modulation in neuropathic pain pathophysiology.

### *Sirt3* deficiency abolished the increased expression ketone synthesis and utilization enzymes by BHB

**Figure 8:**
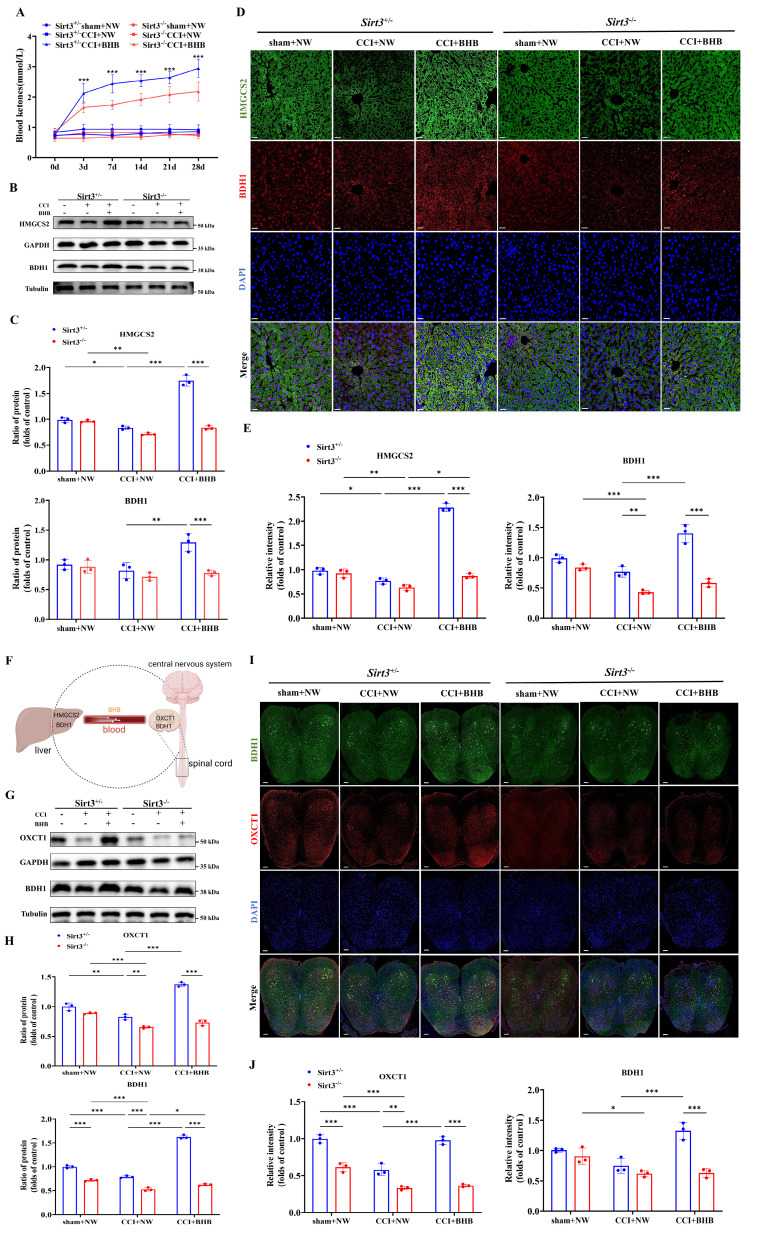
*Sirt3* deficiency reverses BHB-induced expression of BDH1 and HMGCS2 in the liver and BDH1 and OXCT1 in the spinal cord. (A) Blood ketone levels before surgery and at 3, 7, 14, 21, and 28 days post-CCI (n = 5). Statistical comparison: *Sirt3^+/-^CCI+BHB vs. *Sirt3^-/-^*CCI+BHB (*p<0.05, **p<0.01, ***p<0.001). (B) Protein expression of hepatic HMGCS2 and BDH1 at day 7 post-CCI with BHB or saline administration (Tubulin/GAPDH loading controls). (C) Quantification of hepatic ketogenic enzymes (n = 3). (D, E) Immunofluorescence staining of hepatic BDH1 (red) and HMGCS2 (green), counterstained with DAPI (blue). Scale bars: 20 µm (n = 3). (F) Schematic of hepatic ketogenesis and cerebral ketolysis pathways. (G, H) Protein expression of spinal OXCT1 and BDH1 (Tubulin/GAPDH loading controls; n = 3). (I, J) Immunofluorescence staining of spinal cord OXCT1 (red) and BDH1 (green), counterstained with DAPI (blue). Scale bars: 20 µm (n = 3). Data are shown as mean ± SEM. *p < 0.05, **p < 0.01, ***p < 0.001.

Our findings demonstrate that BHB upregulates SIRT3 expression. To assess whether *Sirt3* deficiency affects systemic ketone homeostasis, we measured circulating ketone levels. Notably, genetic ablation of *Sirt3* abolished the BHB-induced elevation of blood ketones (Figure 8A). Given the liver's central role in ketogenesis, we analyzed two key enzymes: β-hydroxybutyrate dehydrogenase 1 (BDH1) and 3-hydroxy-3-methylglutaryl-CoA synthase 2 (HMGCS2). Western blotting revealed that CCI significantly downregulated hepatic HMGCS2 and BDH1 expression. In *Sirt3^+/-^* mice, BHB treatment restored CCI-induced reductions in HMGCS2 and BDH1 expression, consistent with enhanced ketogenic capacity, whereas this effect was absent in *Sirt3^-/-^* mice (Figures 8B and 8C). Immunofluorescence staining further confirmed these hepatic enzyme expression patterns (Figures 8D and 8E), indicating that SIRT3 deficiency impairs BHB-driven ketone synthesis.

Given the spinal dorsal horn's importance in nociceptive processing, we evaluated ketone utilization in this region (Figure 8F). CCI significantly reduced expression of ketolytic enzymes 3-oxoacid CoA-transferase 1 (OXCT1) and BDH1. BHB treatment upregulated OXCT1 and BDH1 in *Sirt3^+/-^* mice after CCI, suggesting enhanced ketone catabolism, but failed to do so in *Sirt3^-/-^* mice (Figures 8G and 8H). Immunofluorescence verified BHB-induced increases in spinal OXCT1 and BDH1, which were abolished by *Sirt3* deletion (Figures 8I and 8J). Together, these results demonstrate that *Sirt3* deficiency disrupts BHB-mediated upregulation of both ketogenic and ketolytic enzymes.

## Discussion

The KD emerges as a multifaceted therapeutic strategy, engaging both metabolic reprogramming and immunomodulatory pathways to combat neuropathic pain 39, 40. By promoting hepatic ketogenesis under carbohydrate restriction, KD generates BHB and acetoacetate—alternative fuels that sustain cerebral and peripheral energy demands while initiating a cascade of neuroprotective effects 41, 42. Our study extends these findings to neuropathic pain pathophysiology by demonstrating that KD alleviates CCI-induced mechanical allodynia and thermal hyperalgesia while suppressing spinal neuroinflammation and microglial hyperactivation. Mechanistically, BHB—the primary effector ketone body of KD—enhances SIRT3 expression to coordinate three therapeutic effects: augmented ketogenesis, mitochondrial functional maintenance, and anti-inflammatory signaling. Strikingly, genetic *Sirt3* ablation fully abrogated KD's neuroprotective benefits, unequivocally establishing SIRT3 as the non-redundant molecular hub for KD-mediated analgesia and neural homeostasis.

In microglia, LPS stimulation activates the Toll-like receptor 4 (TLR4)-mediated signaling cascade, initiating pro-inflammatory pathways (such as NF-κB and MAPK) to drive microglial polarization toward a pro-inflammatory phenotype 43. This activation induces a metabolic shift characterized by enhanced mitochondrial oxidative phosphorylation (OXPHOS) to meet the elevated energy demands for synthesizing inflammatory mediators, thereby increasing the OCR 44, 45. Concurrently, glycolysis is suppressed through dual mechanisms: (1) accumulation of NADH inhibits phosphofructokinase-1 (PFK1), the rate-limiting glycolytic enzyme, and (2) poly (ADP-ribose) polymerase (PARP) activation depletes NAD^+^ pools, reducing glyceraldehyde-3-phosphate dehydrogenase (GAPDH) activity 46-48. While BHB is classically recognized as a mitochondrial fuel that promotes OXPHOS - typically associated with glycolysis suppression (as evidenced by decreased ECAR) 49, 50 - our findings reveal a distinct mechanism under LPS challenge. Mechanistically, despite sustained OXPHOS activation during LPS stimulation, BHB paradoxically alleviates glycolysis inhibition by restoring NAD^+^ availability (via PARP pathway modulation), thereby reactivating glycolytic flux while maintaining OXPHOS activity 51. This dual metabolic reprogramming enables concurrent elevations in both OCR (reflecting OXPHOS) and ECAR (indicating glycolytic recovery), reconciling the apparent contradiction between mitochondrial fuel utilization and glycolytic revival under inflammatory conditions. Future studies are warranted to further elucidate the underlying mechanisms.

Chronic neuroinflammation underpins multiple neurological disorders through sustained activation of microglia and astrocytes 42, 52, 53. The KD demonstrates therapeutic potential by counteracting nociception via glyoxalase 1 activation 54, with BHB serving as the key effector molecule. BHB bypasses metabolic bottlenecks (such as CPT1/PDC) to rapidly fuel mitochondrial β-oxidation 55, 56, while concurrently suppressing NLRP3 inflammasomes, enhancing protein clearance (including α-synuclein), and polarizing microglia toward neuroprotective phenotypes 57-60. However, its clinical translation remains constrained by metabolic state dependency 55.

To investigate the anti-inflammatory and mitochondrial protective mechanisms of BHB, we focused on *Sirt3*, a central mitochondrial deacetylase that regulates microglial activity. SIRT3 coordinates anti-inflammatory and antioxidant responses via PI3K/AKT-SIRT3 and Nrf2/HO-1 pathways 61, 62, while optimizing mitochondrial function through UCP2-mediated ROS scavenging and PGC-1α deacetylation 63, 64. BHB amplifies SIRT3 activity through HDAC inhibition and PPAR/FoxO-dependent transcriptional activation 65-68, synergizing with AMPK-PGC-1α signaling to enhance mitochondrial biogenesis 69, 70. Notably, Sirt3 abolished the suppressive effects of BHB on pro-inflammatory cytokines (TNF-α, IL-6, IL-1β) and disrupted mitochondrial integrity, thereby promoting ROS accumulation and NF-κB-driven inflammatory signaling 71, 72. While BHB modulated UCP2/Pparg/PGC-1α expression independently of *Sirt3* baseline levels, *Sirt3* knockout disrupted BHB-responsive UCP2 activation and systemic ketone homeostasis 69, 70, confirming its indispensability in mediating BHB's effects.

Notably, our study has limitations. First, the precise mechanism by which BHB interacts with the UCP2-SIRT3-PGC-1α signaling axis requires further mechanistic investigation. Second, technical constraints in microglial dynamic imaging and immunostaining resolution necessitate methodological refinements. Finally, the dual role of SIRT3 in regulating hepatic ketogenesis and spinal cord ketone body metabolism warrants further investigation to delineate tissue-specific regulatory mechanisms.

In summary, we establish *Sirt3* as a central regulatory node that functionally connects the anti-neuroinflammatory effects of BHB to mitochondrial integrity and ketone body metabolic homeostasis. These findings position *Sirt3*-targeted therapies and ketogenic interventions as promising strategies for neuropathic pain management.

## Supplementary Material

Supplementary figures and tables.

## Abbreviations

KD: ketogenic diet
CCI: chronic constriction injury
BHB: β-hydroxybutyrate
ROS: reactive oxygen species
UCP2: uncoupling protein 2
SIRT3: sirtuin 3
PGC-1α: peroxisome proliferator-activated receptor-γ co-activator-1ɑ
Pparg: peroxisome proliferator-activated receptor gamma
HMGCS2: 3-hydroxy-3-methylglutaryl coenzyme A synthase 2
BDH1: β-hydroxybutyrate dehydrogenase 1
OXCT1: 3-oxoacid CoA transferase 1
NPP: Neuropathic pain
CNS: central nervous system
HDAC: histone deacetylase
GHRP: growth hormone-releasing peptide
ATP: adenosine triphosphate
HCA: Hierarchical clustering analysis
DEGs: differentially expressed genes
GO: Gene Ontology
KEGG: Kyoto Encyclopedia of Genes and Genomes
TNF-α: tumor necrosis factor-α
IL-6: interleukin-6
IL-1β: interleukin-1β
mtDNA: mitochondrial DNA
OXPHOS: oxidative phosphorylation
qNLRPSPCR: quantitative PCR
NF-κB: nuclear factor kappa-B
GH-IGF-1: Growth Hormone-Insulin-like Growth Factor 1
NLRP3: nucleotide-binding oligomerization domain-like receptor protein 3
DMEM: Dulbecco's Modified Eagle Medium
OCR: Oxygen Consumption Rate
ECAR: Extracellular Acidification Rate
PBS: phosphate-buffered saline
TLR4: Toll-like receptor 4
PFK1: phosphofructokinase-1
PARP: poly ADP-ribose polymerase
GAPDH: glyceraldehyde-3-phosphate dehydrogenase
Nrf2: nuclear factor erythroid 2-related factor 2
Bcl-2: B-cell lymphoma-2
BAX: Bcl2-associated X
ELISA: enzyme-linked immunosorbent assay
BCA: bicinchoninic acid assay
