# Supplementary material for: SIRT3 is required for the protective function of ketogenic diet on neural inflammation and neuropathic pain
Source: Int J Biol Sci. 2025 Apr 21;21(7):3011–29. doi: 10.7150/ijbs.110921 (PMC12080379; doi:10.7150/ijbs.110921)
Supplement: Supplementary file 1 — Supplementary figures and tables. [file ijbsv21p3011s1.pdf]

Supplementary Figure 1 (A) DNA gel electrophoresis to identify *Sirt3*-deficient mice. (B) Western blotting of *Sirt3*$^{+/-}$ and *Sirt3*$^{-/-}$ mice in spinal cord and liver

Supplementary Figure 2

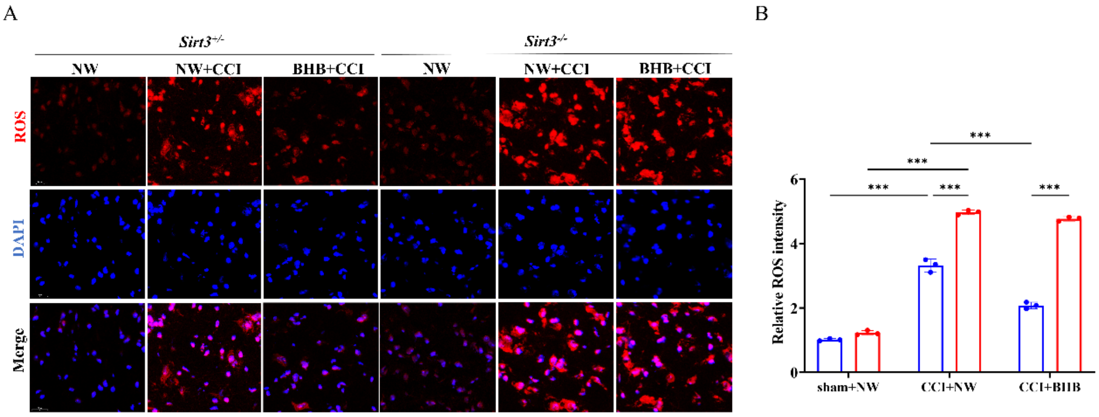

Supplementary Figure 2 (A) Mouse spinal cord ROS fluorescence staining (red), blue for DAPI. (B) Results of ROS fluorescence staining in mouse spinal cord (n=3). Data are shown as mean ± SEM; Two-way ANOVA with Sidak's test for multiple comparisons was performed for all other parameters. $*$ $p < 0.05$, $**p < 0.01$, $***p < 0.001$.

Table S1. Sequences of identification primers for knockout mice.

Gene	Forward primer (5′ → 3′)	Reverse primer (5′ → 3′)
SIRT3 Wild type	CTTCTGCGGCTCTATA CACAG	TGCAACAAGGCTTTATCTTCC
SIRT3 Mutant	TACTGAATATCAGTGGGAACG	TGCAACAAGGCTTTATCTTCC

Table S2. Primer sequences.

Gene	Forward primer (5′ → 3′)	Reverse primer (5′ → 3′)
TNF-α	CGCTCTTCTGTCTACTGAACTTCGG	GTGGTTTGTGAGTGTGAGGGTCTG

IL-6	TTCTTGGGACTGATGCTGGTGAC	CTGTTGGGAGTGGTATCCTCTGTG
IL-1β	CTCGCAGCAGCACATCAACAAG	CCACGGGAAAGACACAGGTAGC
β-actin	TATGCTCTCCCTCACGCCATCC	GTCACGCACGATTTCCCTCTCAG
GAPDH	AGAAGGTGGTGAAGCAGGCATC	CGAAGGTGGAAGAGTGGGAGTTG
UCP2	AAGGTCCGCTTCCAGGCTCAG	ATTACGGGCAACATTGGGAGAAGTC
Pparg	GCCAAGGTGCTCCAGAAGATGAC	GTGAAGGCTCATGTCTGTCTCTGTC
PGC1α	TGAGAATGGATATACTTTACGCAGGTC	TCGTCTGAGTTGGTATCTAGGTCTG
mtDNA	CCTATCACCCTTGCCATCAT-3	GAGGCTGTTGCTTGTGTGAC
ncDNA	ATGGAAAGCCTGCCATCATG	TCCTTGTTGTTCAGCATCAC
ND1	CTAGCAGAAACAAACCGGGC	CCGGCTGCGTATTCTACGTT
